# Y-Osteotomy for Correction of Residual Postoperative Thoracic Kyphotic Deformity in a Patient With Ankylosing Spondylitis

**DOI:** 10.7759/cureus.17633

**Published:** 2021-09-01

**Authors:** Yusuf Omran Hasan, Badryia Toorani

**Affiliations:** 1 Trauma and Orthopaedics, Salmanyia Medical Complex, Manama, BHR

**Keywords:** ankylosing, spondylitis, y osteotomy, correction, deformity

## Abstract

Ankylosing spondylitis (AS) is a chronic inflammatory disease that mainly affects the thoracic spine leading to severe kyphosis. This results in severe functional impairment and sagittal plane imbalance. Surgery is commonly recommended; however, there are many challenges that a spine surgeon may encounter. Several osteotomy techniques have been described to correct these deformities. We describe the use of a relatively novel technique, Y-shape osteotomy, as a surgical option for residual postoperative thoracic kyphotic deformity in a patient with AS with good clinical and radiological outcomes.

## Introduction

Ankylosing spondylitis (AS) is a chronic inflammatory disease that mainly affects the axial skeleton in a caudal to cranial manner [1]. The deformity associated with AS may result in the flattening of the normal lumbar lordosis, thoracic hyperkyphosis, and, in severe cases, forward translation of the head and cervical spine [2]. AS kyphosis is classified into four types: lumbar (type1), thoracolumbar (type 2), thoracic (type 3), and cervical/cervicothoracic (type 5). Thoracolumbar kyphosis deformity (TLKD) (type 2) is the most common type [3].

Aside from the pain and poor cosmetic appearance, the resultant kyphotic deformity leads to severe functional impairment in approximately 30% of patients [4]. This sagittal plane imbalance has been shown to be the most important outcome predictor in spinal deformities [5]. It disturbs the spinal biomechanics, accelerates the adjacent segment degeneration, increases energy expenditure, and increases muscle strain leading to chronic pain [6]. Furthermore, loss of horizontal gaze affects vision in these patients.

To obtain a satisfactory balance in the sagittal or coronal plane, surgery is commonly recommended for AS patients with fixed deformities. Such surgeries on poor, rigid, and brittle bone may pose challenges to spine surgeons [7].

Several osteotomy techniques have been described to achieve a solid fusion of a balanced pain-free spine. Three types are performed, including the Smith-Peterson osteotomy (SPO), pedicle-subtraction osteotomy (PSO), and vertebral column resection (VCR), with each technique having its own indications, limitations, and contraindications.

This paper reports the use of an uncommonly performed technique, the Y-shape osteotomy, as an efficient option for the treatment of residual TLKD in an AS patient who failed initial surgical treatment. We emphasize the value of this technique as a viable alternative to standard osteotomy techniques.

## Case presentation

**Figure 1:**
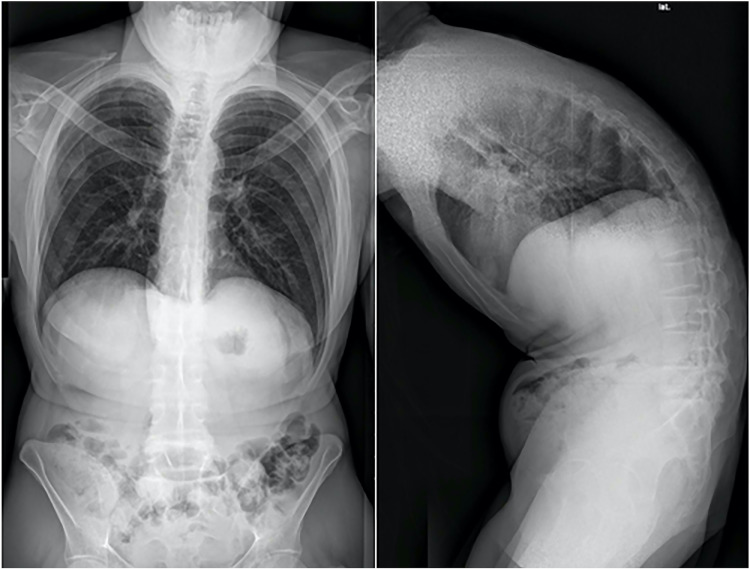
Preoperative x-ray shows TLKD (Type II B+) TLKD, Thoracolumbar kyphosis deformity.

A 40-year-old patient diagnosed with AS by a rheumatologist was referred in 2014 to a spine surgeon due to kyphotic deformity. Records show that the patient reported increasing pain, restriction of movement, and functional impairment in the last few years. As a result, he became drug-dependent. In addition, he is a heavy smoker. On examination, the patient had a humpback and chin-to-chest deformity. On radiological examination, he had thoracolumbar hyperkyphosis and loss of normal lumbar lordosis (Wang Type II B+) (Figure 1). At that time, the operating surgeon decided to do one-level (L4) PSO with T10-S1 fusion. The postoperative course was uneventful. On follow-up, clinical and radiological examination showed incomplete correction of normal kyphosis and sagittal imbalance with residual upper- and mid-thoracic deformity. The decision was to perform a revision surgery.

Surgical note

Preoperative Planning

Anteroposterior and lateral long cassette upright radiograph, computed tomography scan, and magnetic resonance imaging were taken. The level of osteotomy and levels of fusion were selected. We decided to perform a one-level thoracic Y-osteotomy. The patient was optimized medically and reviewed by the anesthetist. Compatible blood units were kept available for transfusion.

Preparing and Positioning

The patient was assumed to have an unstable cervical spine; hence, he was carefully placed in the prone position, avoiding excessive cervical lordosis. Monitoring of somatosensory evoked potentials (SSEPs) and motor evoked potentials was continued, and the possibility of the Stangara wake-up test was explained to the patient and the anesthetist. The level of the osteotomy was aligned with the break of the table.

Operative technique

The decision was to do a Y-shape osteotomy at T8 and extend the fusion to T4 in the prone position with a posterior midline approach. After dissection, free-hand pedicle screws were placed from T4-T7 bilaterally. The spinal canal was decompressed and opened posteriorly at T8 level, and facetectomy was performed in all levels.

**Figure 2:**
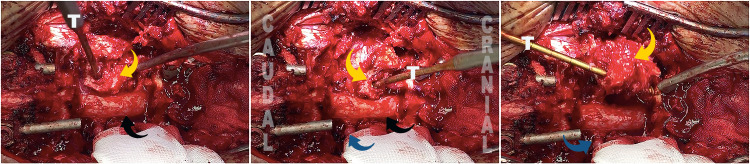
Similar to a lollipop stick, a gentle rotational force was applied to help to extract the pedicles. Pedicle (yellow arrow), spinal cord (black arrow), nerve root (blue arrow), and screw tap (T).

After that, the procedure was carried out as described by Wang [8]. Decancellation procedure of the posterior half of the vertebral body through the pedicle holes (eggshell technique) was performed. The pedicles of T8 were osteotomized, and with the pedicle screw tap inserted acting as a lollipop stick, a gentle rotational force was applied to help extract the pedicles (Figure 2). At this point, a rod was placed temporarily to add stability and avoid anterior translation (sagging), which can compromise the spinal cord or stretch the nerve roots.

**Figure 3:**
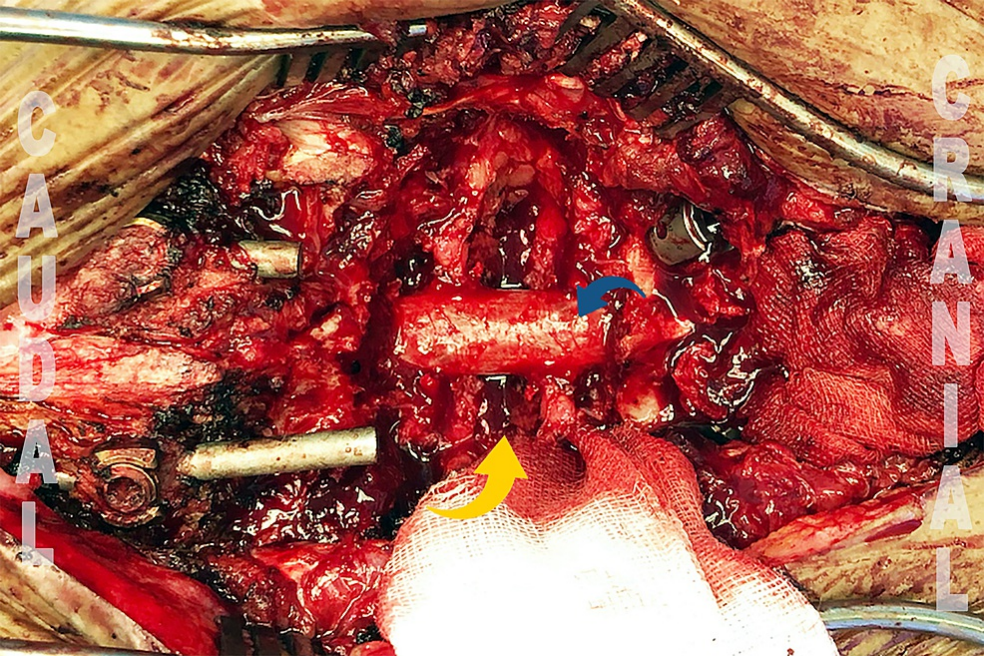
Intraoperative Y-osteotomy and VCD (yellow arrow). Spinal cord (blue arrow). VCD, Vertebral column decancellation.

Afterward, a wedge posterior vertebral cortical osteotomy, thinning and osteotomy of the anterior cortex, and release were carried out as necessary (Figure 3). End-to-end connectors and rods were placed. With alternating gentle distracting and compression forces and the help of the operating table, the posterior wedge was closed, and the anterior column was opened appropriately. Care was taken to detect any kinking or compression of the spinal cord against the caudal or the cranial level during the correction. If so, further decompression was necessary. Finally, the wound was filled with autogenous bone graft and closed in layers.

Intraoperative challenges

The first unanticipated problem was the incomplete calcification of the anterior longitudinal ligament (ALL). In the early stages of AS, the ALL might be not fully calcified. Opening the osteotomy anteriorly may result in osteoclasis through distraction (tension) forces. The preserved elasticity of ligaments can resist such action. This necessitates the release of ALL, which can be challenging for inexperienced hands. Care must be taken not to damage the parietal pleura or accidentally injure the vascular structures anteriorly.

**Figure 4:**
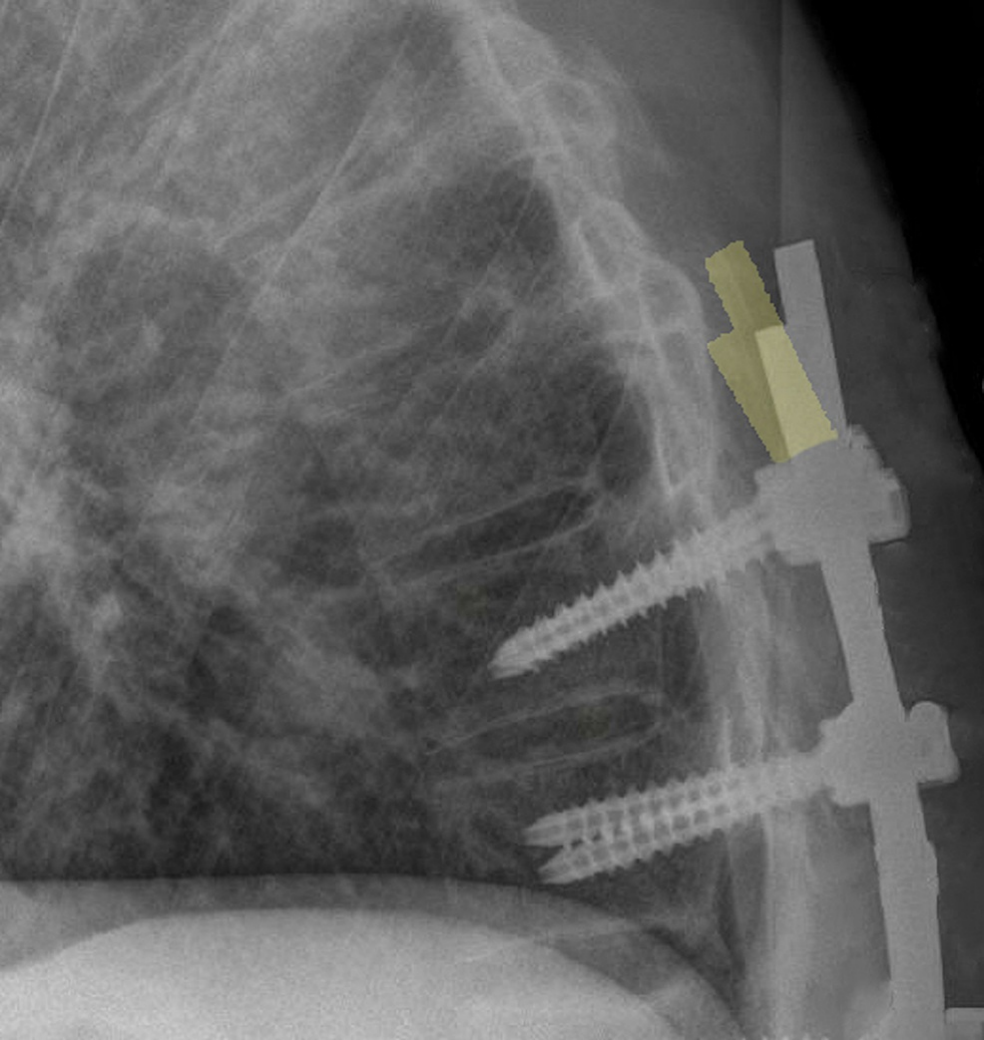
Correction of the old rod orientation proximally (yellow) avoided sagittal subluxation

The second problem was the sagittal (anterior) subluxation of the proximal vertebral column during the correction. This was partially due to the protrusion of the old rod end proximally, which acted as a pivot point. It was detected early and resolved by adjusting the proximal rod end toward the ventral direction (Figure 4).

Results

**Figure 5:**
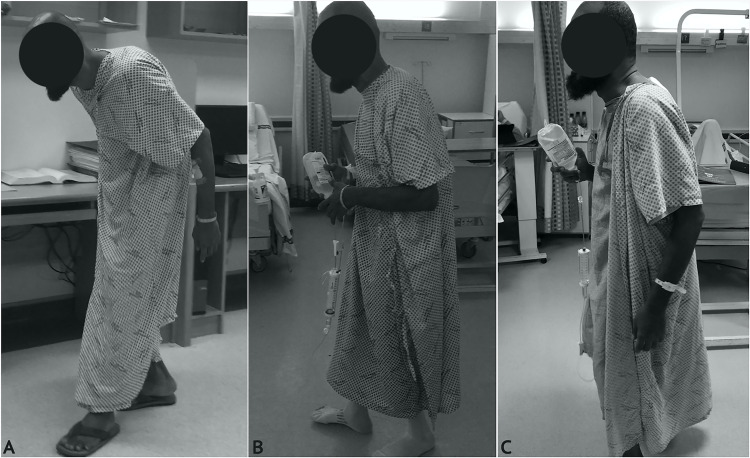
(A) Preoperative. (B) After the first surgery. (C) After the second surgery.

Eighteen months after the second surgery, the patient’s clinical outcome was excellent, with an SRS-22 of 4.3 (range 1-5). He is doing well with high satisfaction (Figure 5).

## Discussion

**Figure 6:**
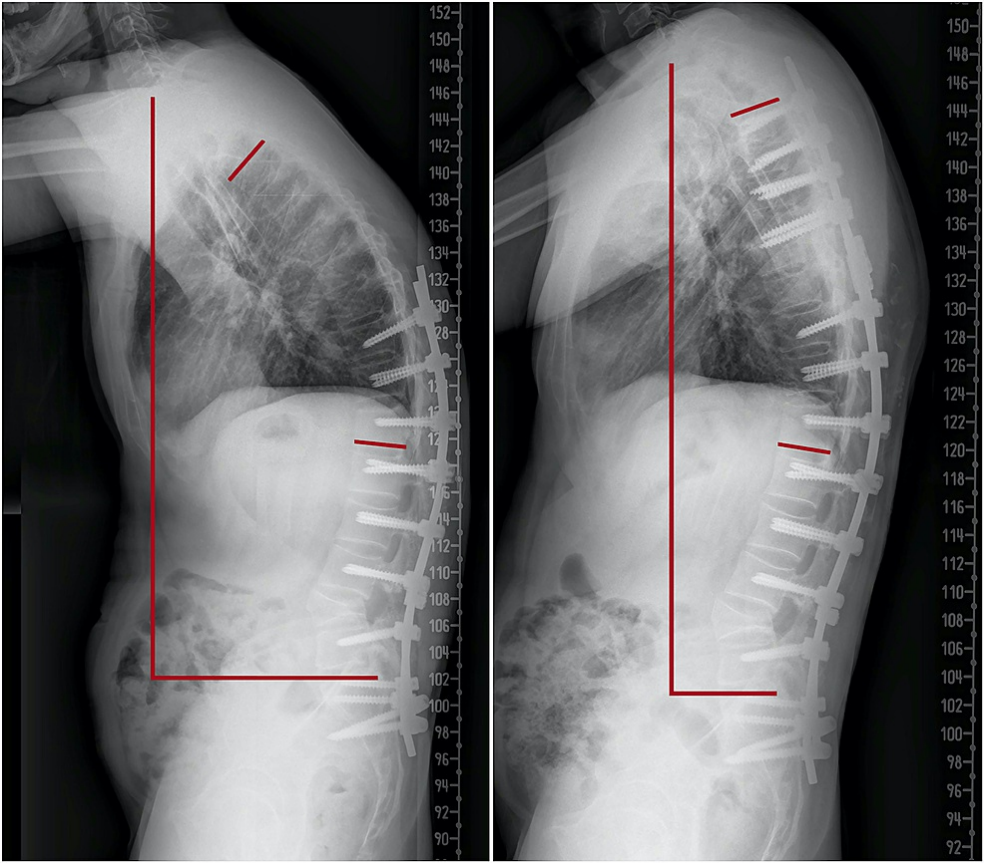
X-rays after the first surgery (left) and second surgery (right) show marked improvement in the sagittal vertical axis and Cobb’s angle (59–31)

Spinal deformity in AS causes global sagittal imbalance, which is usually a fixed deformity. Patients tend to compensate this deformity by flexing the hips and knees. The sagittal vertical axis shifts forward, and the spinopelvic parameters change significantly (Figure 6) [9]. Insufficient correction is a frequent cause of failure and revision surgeries. Decision-making and choice of surgery depend on the number of osteotomies, the number of fixation or fusion levels, location of the osteotomy, and previous surgeries [10]. Therefore, meticulous planning is essential to achieve good clinical and surgical outcomes.

In 1945, Smith-Petersen described his osteotomy (SPO) for the treatment of kyphotic deformity in patients with autoimmune diseases [11]. It is a posterior column osteotomy and is considered a safe, fast, and effective correction tool in treating mild to moderate deformities in the thoracolumbar region, especially if performed in multiple levels. It offers around 10° of correction at each level [12]. The prerequisite of performing this osteotomy is a mobile anterior column and disc. Thus, a fused spine, such as in AS, is considered a contraindication to SPO. In addition, Zhu et al. [13] reported a loss of more than 50° in one-fifth of the patients.

PSO is a V-shaped three-column closing wedge osteotomy usually done at L2 or L3 level. It emerged as a powerful method for deformity correction in the fused spine. It is more demanding but more effective than SPO. One-level osteotomy can result in substantial correction of 30° and between 40° and 50° with the new modifications of the technique [14]. It is ideal for patients with significant positive sagittal imbalance; however, transient neurological deficits occur in 11%─20% of the cases, and significant blood loss has been reported. It is less effective in the thoracic spine (due to shorter vertebral heights) and is associated with a higher complication rate than PSO at the lumbar spine [15].

VCR is used as a last resort for complex rigid deformities when other osteotomies are not feasible. It is used mainly for congenital kyphosis, hemivertebrae, L5 spondyloptosis, and in tumor cases. It is considered the most complex osteotomy with the highest complication and morbidity rates [16].

**Figure 7:**
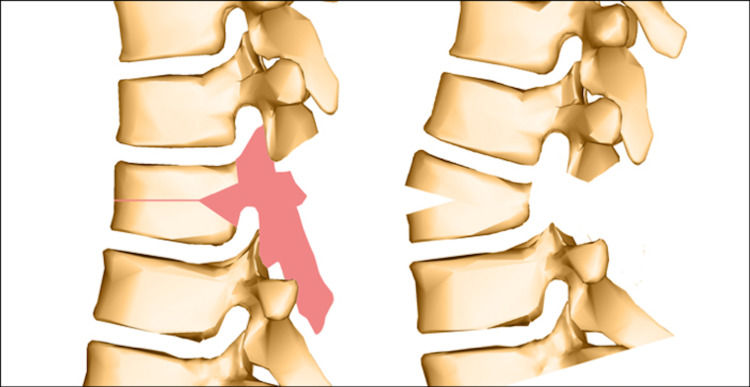
The Y-shaped osteotomy

Recently, a combination of several osteotomy techniques, a Y-shaped osteotomy and vertebral column decancellation (VCD), is considered an alternative useful option. This osteotomy is based on “Y” rather than “V” osteotomy (Figure 7), opening of the anterior column, closing of the posterior column, and a hinge in the middle column. The aim of this technique is to decrease the risk of neural injury by opening and lengthening the anterior column and cortex, decreasing the need to close and shorten the posterior column. Also, the remaining bone of osteotomized vertebra can be utilized in later fusion. In addition, removing as little bone as possible from the middle column enhances the overall stability. Furthermore, the eggshell technique decreases the incidence of vascular injuries.

Wang et al. [17] reported the use of this technique in patients with thoracic kyphosis. His series (45 patients) showed an average of 84% of correction in the immediate postoperative period with no major acute complications or death and no paralysis or implant failure. These results showed that Y-osteotomy and VCD offer safe and reliable ways to achieve good results.

## Conclusions

This osteotomy is still widely unknown, it is less common compared to the other types of spinal osteotomies, and its use in revision surgeries is reported in limited numbers. It is surgically and technically more complex; however, its application is expected to increase in the future because of the excellent and promising early results. It has overcome the limitation of previously known osteotomies achieving a large degree of correction and is proven to be a safe technique.
